# Optimization of Heat Treatment Parameters of AlSi7Mg Alloy

**DOI:** 10.3390/ma15031163

**Published:** 2022-02-02

**Authors:** Jacek Pezda

**Affiliations:** Department of Manufacturing Technology and Automation, University of Bielsko-Biala, 43-309 Bielsko-Biała, Poland; jpezda@ath.bielsko.pl

**Keywords:** Al-Si alloy, mechanical properties, heat treatment

## Abstract

Constantly growing requirements concerning the quality of poured machinery components give rise to the need to find new solutions to improve their mechanical and technological properties, considering economic and ecological aspects resulting from energy consumption of the casting and heat treatment processes. This study presents the investigation of the effect of heat treatment on mechanical properties (tensile strength *R_m_*, elongation *A*_5_, hardness *HBW*) of the AlSi7Mg alloy without modification and modified with strontium. Obtained results allowed us to determine T6 heat treatment parameters associated with the improvement of mechanical properties of the alloy with simultaneous limitation of duration of solutioning and aging treatments. The maximum increase in *R_m_* was 67%, and 55% in the case of *HBW*, with a slight decrease in elongation (approx. 10%) in relation to an alloy not subjected to heat treatment in the adopted scope of tests for the total time of heat treatment of 4–6 h. The increase in elongation of up to 250% requires aging at a temperature exceeding 300 °C, which also causes a decrease in durability and hardness by 20%. Modification of the alloy using strontium before heat treatment facilitates the process of fragmentation and balling of silicone precipitates.

## 1. Introduction

The alloys belonging to the Al-Si group, due to their parameters, are widely used in the aviation and automotive industries. However, very often, they require improvement of their mechanical properties due to high operational requirements for heavily loaded components that must feature a high degree of reliability. An effective method of hardening materials, enabling considerable improvement of their mechanical properties, uses the phenomena of decreasing solubility of alloying elements in the solid-state as the limit temperature decreases [1,2,3,4]. The treatment to which the material is subjected consists of two stages. During the first one (solutioning), the alloy is heated at a temperature above the limit of solubility in the area of a single-phase solid solution and then is rapidly cooled down. During the second one (aging), precipitation of the supersaturating component in the form of finely dispersed precipitates occurs. In the case of alloys containing Cu or Mg, the hardening occurs due to the precipitation of Al_2_Cu Mg_2_Si and Al_2_CuMg phases [1,5,6,7,8].

The treatment used to heat castings in the process of solutioning not only increases the concentration of the elements that are a potential source of precipitation processes in α-Al solid solution but can also cause a favorable change in the morphology of eutectic crystals of silicon–their coalescence and spheroidizing. This improves the mechanical properties of silumins. The form of the eutectics during the heat treatment has a significant effect on the susceptibility of eutectic silicon to coalescence and rounding. The phenomena of coalescence and rounding of the eutectic silicon occur more easily and faster when the particles of eutectic silicon are more dispersed prior to the heat treatment, thus enabling shortened time of heat treatment of the alloy [1,5]. To this end, modifications in a furnace are performed, where micro-additives (modifiers), which impact the morphology of silicon, are added to the liquid alloy. The most commonly used modifiers include Na, Sr, Ca, and Sb [5,9,10,11,12]. Additives of these elements cause changes in the growth of phase *β* (silicon) of the eutectic, which starts to show a more refined morphology, changing its shape from acicular or lamellar to fibrous. Strontium belongs to elements causing a permanent modification of silumins. Strontium is used mainly to modify hypo- and near eutectic alloys, although, according to certain studies [13,14,15,16], its addition brings about positive results also in the case of hyper eutectic alloys, allowing for complex modifications of Si precipitations. In most cases, strontium is inserted into liquid metal in the form of Al-Sr master alloys, containing up to 10% wt. of Sr, and prepared in the form of ingots or extruded rods. The structure of Al-Si eutectics obtained after correct modification is characterized, not only by a minimum interphase distance of the eutectics, but also by rounding of contours and bigger fraction of dendrites of plastic *α*-Al phase compared to an unmodified alloy, which is characterized by large dendrites of *α*-Al phase and lamellar silicon, which look like needles (when viewed in two dimensions). The modifying effect of strontium is analogous to the impact of correct modification using sodium. Moreover, Sr neutralizes the effect of phosphorus and makes the structure of eutectics more fibrous [6,17,18,19,20]. Such a change in the morphology translates into an essential improvement of mechanical properties, in particular ductility, and facilitates the processes of coalescence and coagulation of silicon in the course of heat treatment. In practice, Sr is added within the limits of 0.04–0.07% of the mass of the metallic charge [12].

The duration and temperature of solutioning, suitable cooling, and artificial aging [4,21,22,23] have a direct impact on the improvement of mechanical properties in terms of heat treatment. The ASTM B917-01 standard provides the saturation time of 6–12 h at 540 °C for 356.0 alloys, cooling in warm water, and then 2–5 h of aging at 155 °C, for sand mold castings [24], while in case of metal molds it provides 4–12 h of saturation at 540 °C and 2–5 h of aging at 15 °C [24]. On the other hand, AFS proposes saturation at 538 °C for 12 h and aging at 155 °C for 3–5 h for casting to sand molds, and 227 °C for chill casting molds [25]. Ambient temperature aging processes occurring between quenching and artificial aging (natural aging) should be minimized as they reduce the precipitation driving force necessary for artificial aging [26].

The cost and time of such a type of treatment are of fundamental importance, and the reduction of parameters of the T6 heat treatment process has a beneficial effect on manufacturing costs and productivity [4,27,28,29]. Many authors have carried out studies aimed to determine the effects of parameters of solutioning and aging treatments on mechanical properties and the microstructure of cast AlSi7Mg alloy [30,31,32]. In this area, there are quite large discrepancies in the temperature range and the time of solutioning and aging operations to ensure the required improvement of the mechanical properties of the casting.

The objective of this study was to evaluate the parameters of T6 heat treatment for unmodified and modified AlSi7Mg alloy, taking into account the possibility of improving its mechanical properties, including limitation of the time of the heat treatment with reduction of energy consumption and improved productivity of the process in mind.

## 2. Materials and Methods

Because of its good technological and casting properties, the analyzed alloy is used to manufacture castings for the automotive and machine-building industries. This alloy is poured into sand molds and metal molds and obtains a strength of up to 350 MPa [33,34,35,36,37,38] and up to 400 MPa when fabricated by selective laser melting [39].

In the next step, the studied alloy, featuring the chemical composition presented in Table 1, was melted and refined (Rafal 1, 0.6% mass of metallic charge at the temperature of 730 °C). After refining, the modification was treated via the addition of a modifier in the form of AlSr10 master alloy in the quantity of 0.6% mass of the metallic charge to the bath.

Then, the mold intended for the production of test pieces was filled with refined and modified alloy. The mold was produced in compliance with the PN-88/H-88002 standard [40]. The output cast cylindrical test piece had a diameter of 16 mm and a length of 160 mm.

The heat treatment was performed for unmodified and modified alloy. T6 heat treatment consisted of solutioning heating, followed directly by cooling in cold water (20 °C), and then artificial aging followed by cooling in the open air.

**Figure 1:**
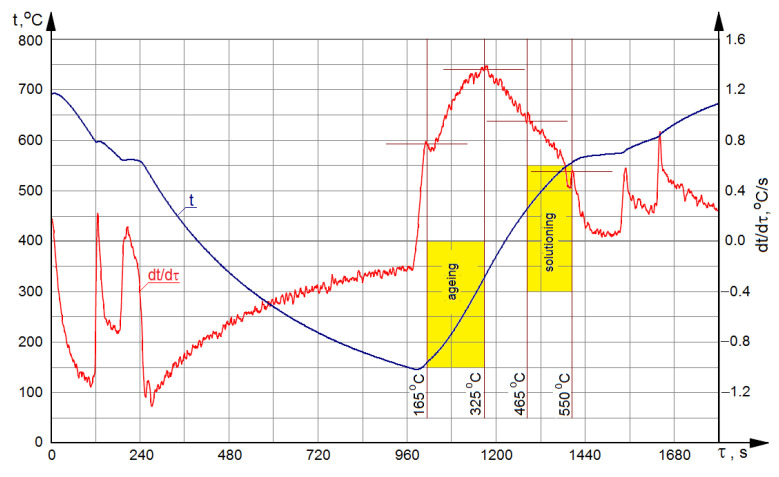
Curves from the DTA method for crystallization and melting of the modified AlSi7Mg alloy.

Figure 1 presents temperature ranges of the solutioning and aging operations as implemented in the course of performed treatments, applied on curves from the DTA method.

The heat treatment of the tested alloy was performed based on a trivalent plan of the testing with four variables (Table 2). For each system from the testing plan, the process was repeated three times, i.e., three test pieces of unmodified alloy and three test pieces of modified alloy were cast and heat treated. Statistic calculations were carried out using Statistica software, version 13.3, developed by StatSoft.

**Figure 2:**
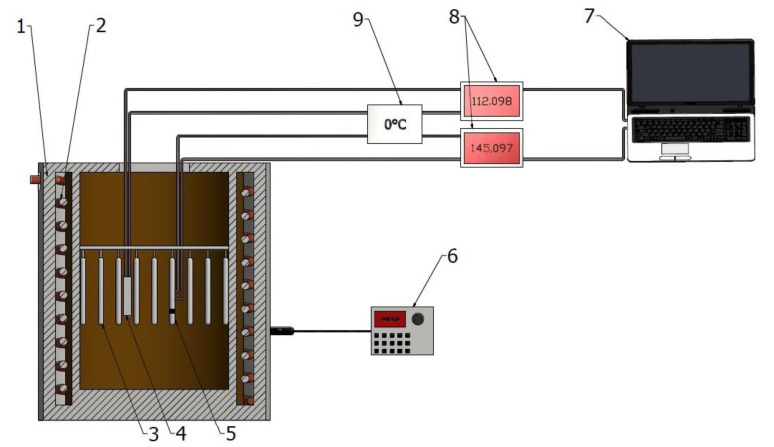
The scheme of the test stand for solution treatment operation (1—electric resistance furnace, 2—heating coil, 3—samples, 4—thermocouple to control furnace’s chamber temperature, 5—thermocouple to control temperature of control sample, 6—furnace control, 7—computer to register the furnace chamber and control sample temperatures (measurement every 15 s), 8—microvoltmeters (accuracy ±0.01 mV), 9—reference thermocouple (kept at 0 °C).

Solutioning and aging (at temperatures exceeding 300 °C) were performed on the testbed; the scheme of the testbed is presented in Figure 2. A resistance furnace constituted the main part of the testbed. To measure the temperature, a type K thermocouple was used, which provided a measuring accuracy at the level of ±5 °C. The thermocouple was inserted into the furnace chamber (allowing measurement of the temperature inside the furnace). The measurement of temperature was carried out simultaneously and continuously. Aging at temperatures below 300 °C was performed in a laboratory dryer of SLN 53 STD type.

The test pieces of the diameter of 10 mm and the measured length of 50 mm, subjected to static tensile strength, were prepared according to ISO 6892-1:2016 standard [41]. The tensile strength test was performed using the Instron 33R4467 tester. The hardness measurement was carried out based on ISO 6506-1:2014 standard [42], using a ball of 10 mm diameter at a load of 9800 N sustained for 30 s. The hardness was measured in the area of milled surfaces of the grasping parts of the test pieces.

The metallographic tests were performed using a Neophot 32 microscope and the “Multiscan” computerized image analysis system.

## 3. Results and Discussion

### 3.1. Tensile Strength R_m_ of the Tested Alloy

**Figure 3:**
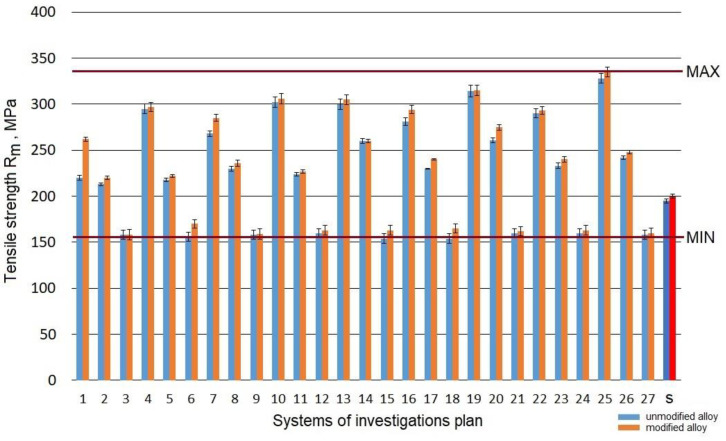
Tensile strength *R_m_* of the studied alloy: s—the initial state.

Raw alloy (without heat treatment) was characterized by tensile strength *R_m_* at the level 190 MPa. The refining resulted in the growth of the strength to 195 MPa, while the modification made it possible to obtain a value of 200 MPa. The performed heat treatment resulted in a change of the tensile strength *R_m_* from 154 to 328 MPa for an unmodified alloy and from 154 to 335 MPa for a modified alloy. Taking into account the results obtained in the assumed scope of the tests for the refined and modified alloy before and after the heat treatment, a considerable increase in strength *R_m_* (Figure 3) was obtained for the systems from the testing plan denominated as: no. 25 (*t_s_* = 550 °C; *τ_s_* = 3 h; *t_a_* = 165 °C; *τ_a_* = 8 h), no. 13 (*t_s_* = 520 °C; *τ_s_* = 1.5 h; *t_a_* = 165 °C; *τ_a_* = 5 h) and no. 19 (*t_s_* = 550 °C; *τ_s_* = 0.5 h; *t_a_* = 165 °C; *τ_a_* = 5 h). A similar strength at the level 300 MPa was recorded for systems no. 4, 10, 16, which is typical for a low aging temperature (165 °C). On the other hand, the lowest tensile strength *R_m_* was obtained for the temperature of aging t_a_ = 325 °C (systems no. 3, 9, and 27).

**Figure 4:**
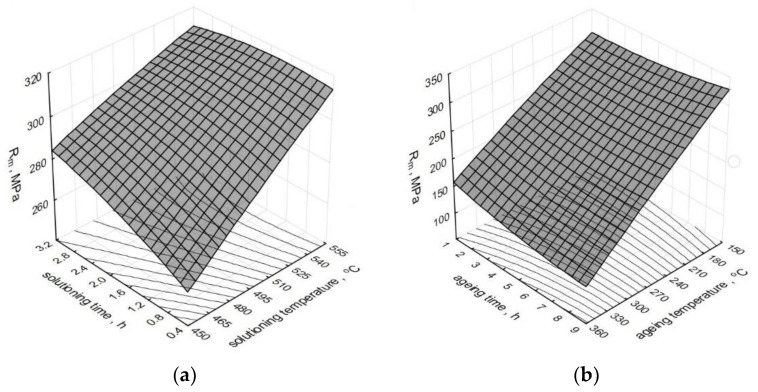
Effect of T6 heat treatment parameters on the tensile strength *R_m_* of the tested alloy: (**a**) *t_s_* and *τ_s_*; (**b**) *t_a_* and *τ_a_*.

The effect of the heat treatment operations’ parameters on the change of tensile strength *R_m_* of the modified alloy is depicted by spatial diagrams (Figure 4). The spatial diagrams were prepared based on a regression equation in the form of the second-degree polynomial, which takes the form of the relation (1). The equation significance is *α* = 0.05.

(1) $$R_{m}=-64.66+0.738t_{s}+2.53\cdot 10^{-4}t_{s}^{2}+60.855\tau_{s}-0.844\tau_{s}^{2}+0.655t_{a}-2.41\cdot 10^{-4}t_{a}^{2}+7.64\tau_{a}+0.364\tau_{a}^{2}-0.105t_{s}\tau_{s}-22.68\cdot 10^{-4}t_{s}t_{a}-0.007t_{s}\tau_{a}-0.027\tau_{s}t_{a}+0.805\tau_{s}\tau_{a}-0.036t_{a}\tau_{a}\;$$

where: *t_p_*—solutioning temperature, *τ_p_*—solutioning time, *t_s_*—aging temperature, *τ_p_*—aging time. Correlation coefficients: R^2^ = 0.98; corrr. R^2^ = 0.96.

For the unmodified alloy, the dependency (1) takes the form (2):

(2) $$R_{m}=-573.6+1.7t_{s}-0.1\cdot 10^{-4}t_{s}^{2}+111.08\tau_{s}-2.904\tau_{s}^{2}+1.648t_{a}+5.15\cdot 10^{-4}t_{a}^{2}+37.74\tau_{a}+0.008\tau_{a}^{2}-0.166t_{s}\tau_{s}-36.4\cdot 10^{-4}t_{s}t_{a}-0.047t_{s}\tau_{a}-0.051\tau_{s}t_{a}-0.388\tau_{s}\tau_{a}-0.053t_{a}\tau_{a}\;$$

Correlation coefficients: R^2^ = 0.97; corr. R^2^ = 0.95.

As constant values for the solutioning, we assumed time of *τ_s_* = 1 h and temperature of *t_s_* = 520 °C, while in case of the aging: time of *τ_a_* = 5 h and temperature of *t_a_* = 165 °C.

Solutioning the alloy for 1 to 2 h at temperature t_s_ = 540–550 °C and aging it at temperature *t_a_* = 165 °C for 5 to 8 h causes a significant increase in the tensile strength *R_m_*. By solutioning the alloy modified at the temperature of 540 °C for 1 h and aging it at the temperature of 180 °C for one and four hours, Moller et al. [43] obtained *R_m_* = 303 MPa, while the further increase obtained at the level of 10% resulted from the content of Mg in the alloy increased to 0.4%. By solutioning the alloy at the same temperature for one hour longer and by aging it at 170 °C for 7 h, the authors of the study [44] obtained the strength of 306 MPa with an addition of 0.5% Ti. The authors of the study [45] obtained a similar strength (328 MPa) after solutioning at 535 °C for 6 h and aging at 160 °C for 8 h at the initial value *R_m_* of the untreated alloy at the level of 252 MPa, i.e., approximately 25% greater compared to the presented test results. Pedersen [46] obtained the maximum strength at 60 min of solutioning at 540 °C and 4 h of aging at 150 °C, and the longer time of treatment did not increase the strength. Ragab et al. [47] obtained UTS at the level exceeding 300 MPa for an unmodified alloy and a modified alloy after solutioning at 535 °C for 5 h and aging after 24 h at 170 °C for 4, 8, and 12 h.

According to Shivkumar [48], solutioning for 3–6 h at the temperature of 540 °C is optimal for a modified alloy cast to sand molds. In his study, Zhang [49], after 30 min of solutioning at the temperature of 540 °C and 550 °C, obtained more than 90% of R_0.2_ value and more than 95% of *R_m_* was obtained using standard solutioning for 6 h. Peng [50] achieved similar mechanical properties, however, after solutioning at 550 °C for 2 h and aging at 170 °C, also for 2 h. Reduction of the aging time below 5 h, assuming the aging temperature below 180 °C is maintained, should provide strength at the level of 300 MPa (Figure 3) and 90–95% of *R_m_* value obtained for aging exceeding 5 h, as in the case of work [50], where reduction of the aging time from 15 to 2 h decreased the tensile strength by merely 8 MPa.

### 3.2. Elongation A_5_ of the Tested Alloy

**Figure 5:**
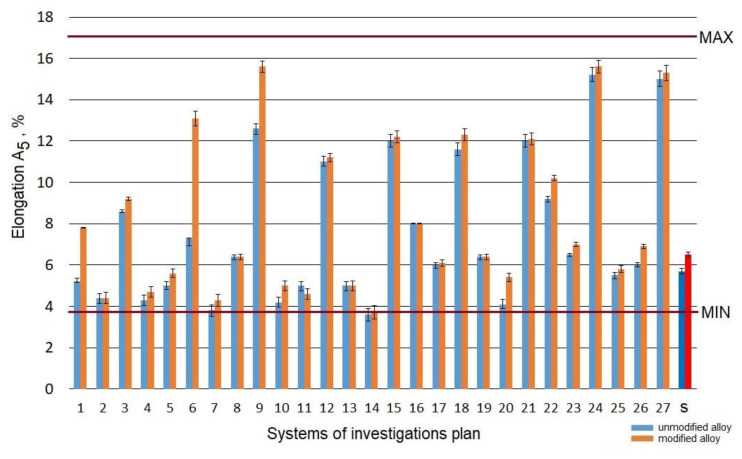
Elongation *A_5_* of the studied alloy: s—the initial state.

The raw alloy (without the heat treatment) was characterized by elongation *A_5_* at the level of 5.8% in the case of the refined alloy and 6.5% in the case of the modified alloy. The performed heat treatment resulted in a change of the elongation, which was included within the range of 3.7–15.6% (Figure 5).

Considering the results obtained in the assumed area of the testing plan for refined and modified alloy before and after the heat treatment, the elongation *A_5_* increased in the case of systems no. 9 (*t_s_* = 465 °C; *τ_s_* = 0.5 h; *t_a_* = 325 °C; *τ_a_* = 8 h), no. 24 (*t_s_* = 550 °C; *τ_s_* = 1.5 h; *t_a_* = 325 °C; *τ_a_* = 5 h), and no. 24 (*t_s_* = 550 °C; *τ_s_* = 1.5 h; *t_a_* = 325 °C; *τ_a_* = 5 h). A high aging temperature (325 °C) results in an increase in elongation of the alloy, with a simultaneous decrease in its strength. This is due to over-aging of the alloy and results from the loss of coherence of the phase precipitated with the alloy’s matrix, as well as coagulation of particles of eutectic silicon.

Aging at low temperature (*t_a_* = 165 °C) for 5–8 h causes a decrease in the elongation *A_5_* (systems no. 4, 7, 10, and 14) compared to the alloy not subjected to heat treatment, both modified and unmodified, by 10–20%. This results directly from the hardening of the alloy with small precipitates of Mg_2_Si particles inside *α*-Al grains [51]. In addition, reduction of the elongation of the alloy after heat treatment is caused by the direct effect of coarsening of Si particles after T6 temper [52] and is more influenced by the cracking mechanism acting within the material [53].

**Figure 6:**
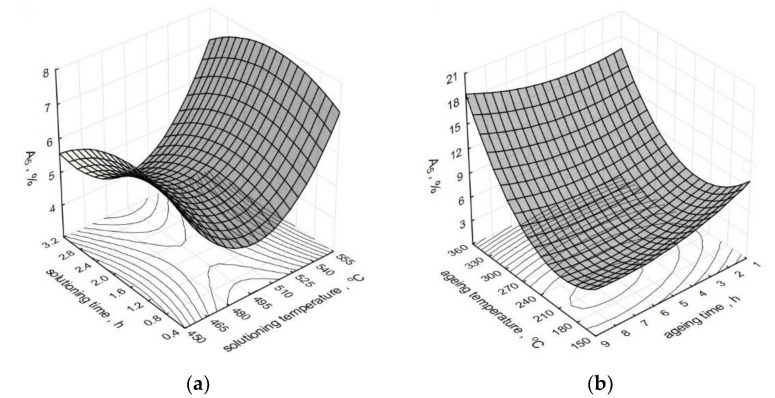
Effect of T6 heat treatment parameters on elongation *A_5_* of the studied alloy: (**a**) *t_s_* and *τ_s_*, (**b**) *t_a_* and *τ_a_*.

The effect of parameters of the heat treatment operations on the change of the elongation A_5_ of the modified alloy is depicted by spatial diagrams (Figure 6). The spatial diagrams were prepared based on a regression equation in the form of the second-degree polynomial, which takes the form of the relation (3). The equation significance is *α* = 0.05.

(3) $$A_{5}=234.75-0.789t_{s}+7.94\cdot 10^{-4}t_{s}^{2}-1.43\tau_{s}-0.382\tau_{s}^{2}+0.269t_{a}+5.83\cdot 10^{-4}t_{a}^{2}-2.1\tau_{a}-0.087\tau_{a}^{2}+0.002t_{s}\tau_{s}-0.17\cdot 10^{-4}t_{s}t_{a}+0.003t_{s}\tau_{a}+0.009\tau_{s}t_{a}+0.012\tau_{s}\tau_{a}+0.003t_{a}\tau_{a}\;$$

where: *t_p_*—solutioning temperature, *τ_p_*—solutioning time, *t_s_*—aging temperature, *τ_p_*—aging time. Correlation coefficients: R^2^ = 0.97; corrr. R^2^ = 0.94.

For the unmodified alloy, the dependency (3) takes the form (4):

(4) $$A_{5}=110.12-0.331t_{s}+3.43\cdot 10^{-4}t_{s}^{2}-2.13\tau_{s}-0.163\tau_{s}^{2}-0.271t_{a}+4.8\cdot 10^{-4}t_{a}^{2}+0.282\tau_{a}+0.043\tau_{a}^{2}-0.005t_{s}\tau_{s}+0.85\cdot 10^{-4}t_{s}t_{a}-0.003t_{s}\tau_{a}+0.005\tau_{s}t_{a}-0.117\tau_{s}\tau_{a}-0.004t_{a}\tau_{a}\;$$

Correlation coefficients: R^2^ = 0.9; corr. R^2^ = 0.79.

As constant values for solutioning, we assumed time *τ_s_* = 1 h and temperature *t_s_* = 520 °C, while in case of aging: time *τ_a_* = 5 h and temperature *t_a_* = 165 °C.

Obtaining the maximum increase in the elongation *A_5_* of the alloy is preconditioned by its aging at a temperature above 300 °C for 5–8 h after previous solutioning for 30–90 min at the temperature of 520–550 °C. Considering the fact that the temperatures of aging treatment of the AlSi7Mg alloy practically do not exceed 200 °C (in most cases, they fluctuate between 150–180 °C), the obtained elongation was at the level of 6–7%. A similar value of the elongation (7%) was obtained by Liu [45] after 6 h of solutioning at 535 °C and 2 h of aging at 180 °C. Twice as long aging time at the temperature of 520 °C and 8 h of aging at 160 °C enabled Zyska [54] to achieve 8% elongation for the unmodified alloy and even 11% for the alloy subjected to modification. Elongation at the level 5.3% required 8 h of solutioning at 530 °C and 6 h of aging at 180 °C, while in the case of the author of the paper [32], after solutioning of the alloy at 530 °C for 8 and 5 h. Three hours shorter time of solutioning at the same temperature and aging at 170 °C for 1–8 h allowed to obtain elongation of a modified alloy between 4.2 and 5% [47]. Much lower elongation (3%) after reduction of solutioning time to 2 h and 2 h of aging at 170 °C is reported by the author of the paper [55]. An increase in solutioning temperature to 540 °C resulted in a decrease in elongation within the limit of 10%, same as in the case of the paper [31], where 4 h of solutioning at this temperature and 6 h of aging at 160 °C resulted in 3.2% elongation for metal mold and 1% for the casting from a sand mold. Much higher elongation, i.e., 5%, for an unmodified alloy and 7% for a modified alloy, was obtained by Wang [52] after solutioning at the same temperature for 12 h and aging at 155 °C for 10 h. The use of the same solutioning temperature allowed another author [30] to obtain 8.7% elongation after 6 h of solutioning and 4 h of aging at 155 °C. Lu [28] limited the solutioning time to 30 min (aging for 90 min at 180 °C); the result accounted for 90% of the elongation value observed in the case of 10 h of solutioning at the same temperature and 5 h of aging at 170 °C. Similarly, Zhang [56], for a low-pressure casting, obtained 95% of the maximum elongation after solutioning for 6 h at 540 °C. However, Peng achieved 80% of the maximum elongation (7%) obtained after solutioning at 535 °C for 4 h and aging at 170 °C for 15 h [50] after 2 h of solutioning at 550 °C and aging at 170 °C. Taking into accout the elongation after heat treatment of modified and unmodified alloys, it should be ascertained that modification has a positive effect on elongation of the untreated alloy [54,57] due to refining of silicon precipitates and that it facilitates their spheroidization during T6 treatment [58]. Moreover, spheroidization of silicon precipitates results in the growth of fracture toughness and ductility [59,60]. Taking into account the high hardness and low ductility of the precipitates of eutectic silicon, the growth of their size causes a reduction in the fracture stress of A356 alloys. At the same time, the decrease in the ultimate tensile strength and a slight increase in ductility can be noticed [61].

### 3.3. Hardness HBW10/1000/30 of the Tested Alloy

**Figure 7:**
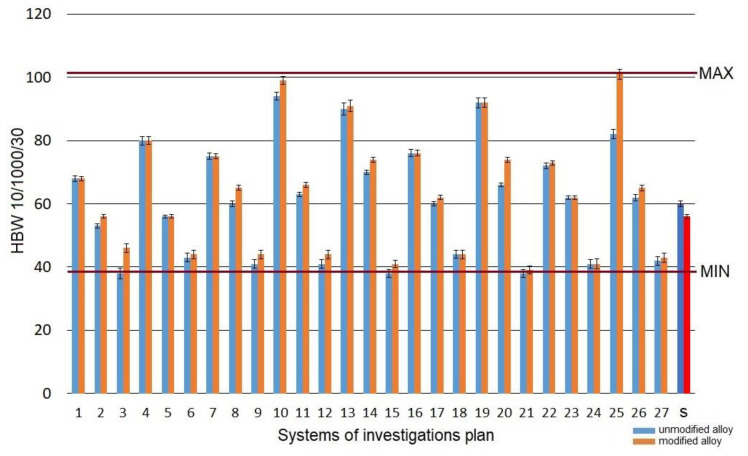
Hardness *HBW10/1000/30* of the studied alloy: s—the initial state.

As-cast alloy (without heat treatment) featured hardness at the level of 58 *HBW10/1000/30* for modified alloy and 60 *HBW10/1000/30* for unmodified alloy. Heat treatment operations changed the hardness of the alloy within the range of 41–101 *HBW10/1000/30* (Figure 7).

The highest increase in hardness *HBW* was confirmed for the system marked as no. 10 (*t_s_* =520 °C; *τ_s_* =30 min; *t_a_* =165 °C; *τ_a_* =8 h)—99 *HBW10/1000/30* and marked as no. 25 (*t_s_* = 550 °C; *τ_s_* =30 min; *t_a_* =165 °C; *τ_a_* =8 h)—101 *HBW10/1000/30*. The test pieces from the system no. 13 (*t_s_* =520 °C; *τ_s_* =90 min; *t_a_* =165 °C; *τ_a_* =5 h) and from the system no. 19 (*t_s_* =550 °C; *τ_s_* =30 min; *t_a_* =165 °C; *τ_a_* =5 h) are characterized by a slightly lower hardness, i.e., 91–92 *HBW10/1000/30*. The lowest hardness was obtained for the systems nos. 6, 9, 12, 15, 24, for which the aging temperature was 325 °C regardless of the time of this operation. The obtained hardness was at the level of 41–44 *HBW10/1000/30*, which denotes a significant decrease in relation to the initial alloy.

**Figure 8:**
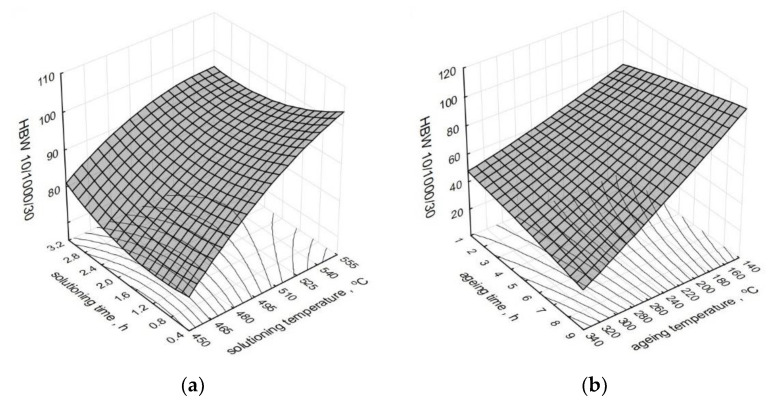
Effect of the heat treatment parameters on the hardness *HBW10/1000/30* of the studied alloy: (**a**) *t_s_* and *τ_s_*, (**b**) *t_a_* and *τ_a_*.

The effect of the heat treatment parameters on the change of the hardness *HBW10/1000/30* of a modified alloy is presented in the form of spatial diagrams (Figure 8). The spatial diagrams were created based on the regression equation in the form of a polynomial of the second degree, which takes the form of the relation (5). The equation significance is 𝛼 = 0.05.

(5) $$HBW=-577.98+2.277t_{s}+18.02\cdot 10^{-4}t_{s}^{2}+17.168\tau_{s}+1.363\tau_{s}^{2}+0.44t_{a}+2.39\cdot 10^{-4}t_{a}^{2}+2.93\tau_{a}-0.043\tau_{a}^{2}-0.049t_{s}\tau_{s}-13.73\cdot 10^{-4}t_{s}t_{a}+0.008t_{s}\tau_{a}+0.009\tau_{s}t_{a}+0.073\tau_{s}\tau_{a}-0.02t_{a}\tau_{a}\;$$

where: *t_s_*—solutioning temperature, *τ_s_*—solutioning time, *t_a_*—aging temperature, *τ_a_*—aging time. Correlation coefficients: R^2^ = 0.98; corrr. R^2^ = 0.95.

For the unmodified alloy, the dependency (5) takes the form (6):

(6) $$HBW=-636.31+2.661t_{s}-23.01\cdot 10^{-4}t_{s}^{2}+28.184\tau_{s}-0.252\tau_{s}^{2}+0.04t+3.2\cdot 10^{-4}t_{a}^{2}+5\tau_{a}-0.222\tau_{a}^{2}-0.064t_{s}\tau_{s}-7.72\cdot 10^{-4}t_{s}t_{a}+0.005t_{s}\tau_{a}+0.027\tau_{s}t_{a}-0.256\tau_{s}\tau_{a}-0.02t_{a}\tau_{a}\;$$

Correlation coefficients: R^2^ = 0.98; corr. R^2^ = 0.96.

As constant values for the solutioning, we assumed time *τ_s_* = 1 h and temperature *t_s_* = 520 °C, while in the case of the aging: time *τ_a_* = 5 h and temperature *t_a_* = 165 °C.

Solutioning of the tested alloy in the temperature range from 525 to 540 °C for 30 to 60 min and aging at the temperature of 165 °C for 5 to 8 h allowed to obtain the highest values of hardness *HBW10/1000/30* for both unmodified and modified alloy. The authors of the paper [61], after solutioning at the temperature of 540 °C for 75 min and aging at the temperature of 170–180 °C for 10 h, obtained hardness at a similar level (100 HB). By prolonging the time of solutioning to 8 h and aging A356 alloy for 6 h at 160 °C, the authors of the study [62] obtained hardness of 118 *HV* after the addition of 0.4% wt. of Sc. The hardness within the range of 111–116 *HB* was obtained by Cechini et al. [63] by solutioning A356 alloy for 4.5 h at the temperature of 535 °C and artificially aging it at 160 °C for 4.5 h. In the case of the components manufactured in the semi-solid state, to obtain hardness at the level of 125 *HBW*, the authors of the study [64] performed the solutioning for only 15 min at 540 °C and aging at 180 °C for 3 h. The obtained values correspond to the values of the hardness obtained after 8 h of solutioning (conventional heat treatment according to ASTM B 917 standard). Both for modified and unmodified alloy, the obtained hardness is directly connected with the temperature of the aging. Aging at the temperatures of up to 180 °C (peak temperature) increases the hardness, while at the temperature above 200–220 °C, a decrease in the hardness can be observed as a result of over-aging the alloy [65]. The hardness is mainly controlled by the microstructure containing coherent rods or needles of *β*’ (Mg_2_Si). When aging at 180 °C, coherent with a matrix of Mg_2_Si (*β*’), needles have an impact on the increase in the hardness. Whereas, at the temperature of 220 °C, precipitation of stable, equilibrium phase *β* (Mg_2_Si) takes place, which is incoherent with the matrix. In such a case, the hardness tends to decrease upon the growth of the aging temperature. Thus, changes in the hardness resulting from the use of different aging temperatures in the course of heat treatment are directly correlated with Mg_2_Si precipitation [66]. Metastable intermediate fases *β*’ and *β*” are responsible for the hardening of the alloy, and hence, for the increase in the hardness.

### 3.4. Microstructure

**Figure 9:**
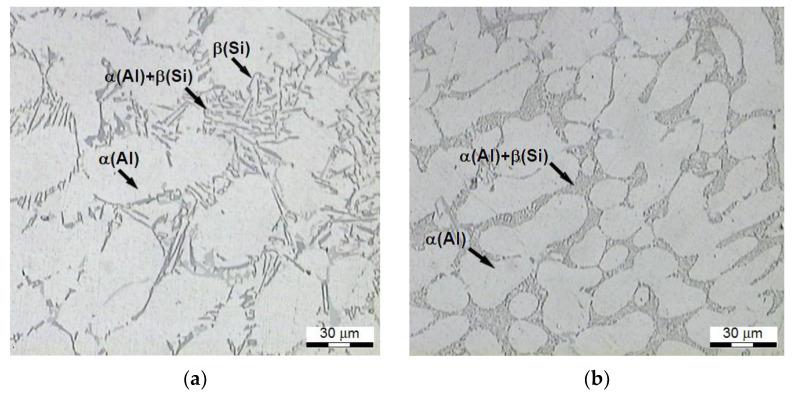
Microstructure of AlSi7Mg alloy in the initial state after: (**a**) refining, (**b**) refining and modification.

The microstructure of AlSi7Mg alloy, modified and unmodified, before its heat treatment, is presented in Figure 9.

The refined alloy is characterized by lamellar silicon eutectics, shaped in the form of needles (Figure 9a) located within interdendric spaces, while the microstructure after the modification (Figure 9b) is characterized by a minimal interphase distance of the eutectics with fine and fibrous precipitates of Si, and with roundings of contours of large dendrites of plastic *α*-Al phase [67,68]. The Si precipitates visible in the photos of microstructures do not constitute separate, spherical crystals, but these are cross-sections of thin branchings of bigger eutectic Si crystals with developed dendritic or coral structures [52].

**Figure 10:**
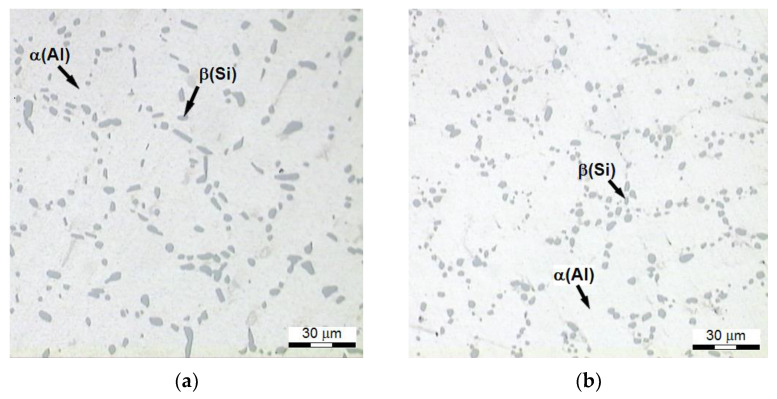
Microstructure of AlSi7Mg alloy is characterized by the highest strength *R_m_*: (**a**) refined alloy (328 MPa), (**b**) refined and modified alloy (335 MPa).

As a result of the performed heat treatment, the silicon is subjected to three successive processes: decomposition, spheroidization, and coarsening [69], and the holding time increases [70]. Microstructures (Figure 10) of the alloy featuring the highest tensile strength *R_m_* (system no. 25, Figure 2) have distinctive and nearly spheroidal (Figure 10b) Si precipitates within interdendric areas of phase Al. In the case of the alloy after modification, rounded corners of Si precipitates in the case of refined alloy (Figure 10a) were created after partial defragmentation of the bigger precipitates of Si. The strength and the ductility are improved by small, nearly spherical, eutectic precipitates of silicon, which are resistant to plastic deformations, and which restrict nucleation of cracks [56,70,71]. While fine eutectic particles of the silicon have a positive effect on the strength, their thickening reduces it. It is especially noticeable in the case of structures of over-aged alloy. Thus, the evolution of the eutectic silicon starts from disintegration and spheroidization of the eutectic silicon resulting from striving of the system (alloy) to minimize its free energy in given conditions by reduction of overall separation area of the phases, after which, together with the increase in time of the treatment, coarsening of the eutectic silicon, caused by the supply of Si atoms diffusing out of cores of dendrites, as well as by Ostwald ripening mechanism [70,72] occur. In the light of the obtained results, the form of Si precipitates does not have a significant effect on the tensile strength *R_m_* of heat-treated alloy. This can be confirmed by results obtained by the authors of the study [54], where the *R_m_* strength in case of the modified alloy after solutioning treatment at 520 °C for 12 h and aging for 8 h at 180 °C was higher by only 16 MPa compared to the unmodified alloy (215 MPa) treated in the same conditions. Wang [52], after solutioning an unmodified and a modified alloy at 540 °C for 6 h and aging them at 155 °C for 4 h, obtained a difference between them at a level of 30 MPa. A similar difference was obtained by Emamy [30] after solutioning at the same temperature for a twice longer time and aging for 6 h longer at 155 °C.

**Figure 11:**
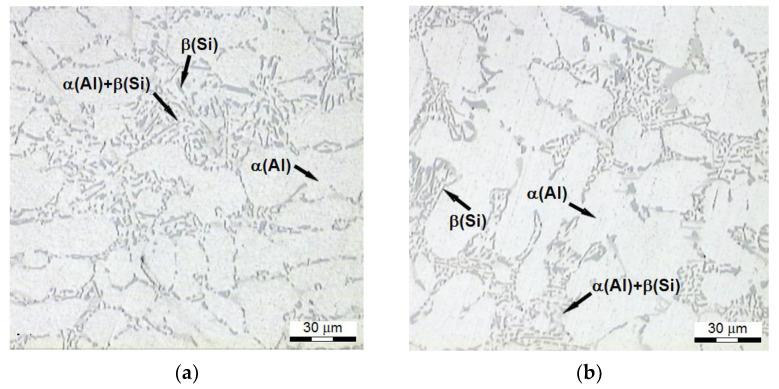
Microstructure of AlSi7Mg alloy, which is characterized by the lowest *R_m_* strength: (**a**) refined alloy (158 MPa), (**b**) modified alloy (159 MPa).

Microstructure (Figure 11) of the alloy of the test pieces having the lowest tensile strength *R_m_* (system no. 3-Figure 2) features extended dendrites of phase Al with the eutectic similar to the modified alloy, without distinct Si precipitates characteristic for heat-treated alloy. It is connected with a temperature too low compared to the temperature of conventional solutioning (465 °C) and the short time of this operation (30 min). Silicon atoms diffuse more easily at higher temperatures (550 °C); thus, eutectic particles of the silicon may undergo a morphological transformation during a shorter time of solutioning at this temperature.

It was confirmed by Peng [50], who obtained full spheroidization of Si particles and oversaturation of Si and Mg in α-(Al) after 4 h of solutioning at 535 °C for a half shorter time at the temperature of 550 °C. Rometsch [59] has demonstrated that only 35 min of solutioning was sufficient for the complete dissolution of Mg_2_Si and homogenization of Mg. Whereas, according to Zhang [49], 10 min of solutioning at the temperature of 540 °C or 550 °C is sufficient to obtain the maximum level of Mg and Si, which results from limiting the solubility and chemical composition of the alloy. However, prolongation of the treatment time to 30 min ensures spheroidization of precipitates of eutectic silicon and increases the space between these precipitates. As a result, the impact strength and ductility of the alloy, spheroidization of eutectic silicon, and dissolution of Mg_2_Si phase after 30 minutes of solutioning at 540 °C are improved, which is also confirmed by the study [28]. Such a short solutioning time applies to modified alloys, for which it is not necessary to dissolve branches of the silicon skeleton, characteristic of the unmodified alloy. As a result, quick spheroidization and coarsening can be obtained due to the use of shorter alloy solutioning times [56].

## 4. Conclusions

The obtained test results allowed us to evaluate ranges of solutioning and aging treatment parameters required to improve mechanical properties of AlSi7Mg alloy refined and modified with strontium.

It is possible to considerably shorten the time of the solutioning and aging, thus reducing energy consumption and improving the productivity of the process while maintaining the required mechanical properties of the AlSi7Mg alloy at the same time.

Performing modification of the AlSi7Mg alloy prior to its heat treatment does not have any significant effect on the obtained tensile strength and hardness; however, this facilitates fragmentation of silicon precipitates and their spheroidization during T6 treatment, which in turn leads to significant improvement in ductility and impact strength of the alloy.

The spatial diagrams illustrate the effect of the heat treatment parameters on selected mechanical properties (tensile strength *R_m_*, elongation *A_5_*, hardness *HBW*) obtained on the base of the assumed area of the testing.

Properly selected temperatures and times of solutioning and aging have a significant effect on the quality of the alloy; therefore, they determine the reliability of components of machinery and devices produced from the alloy to a high degree.

Obtaining tensile strength *R_m_* at the level of 320 MPa and hardness of up to 100 *HBW/10/100/30* require solutioning of the alloy for 30 to 90 min at the temperature of 540–550 °C followed by aging for 5–8 h at 165 °C (with the possibility of its shortening to 2–3 h). Whereas in the case of elongation *A_5_*, the main parameter having an effect on its increase is an aging temperature exceeding 300 °C.

## Figures and Tables

**Table 1:** Chemical composition of EN AC–AlSi7Mg alloy, wt. %.

Si	Fe	Cu	Mn	Mg	Cr	Ni	Zn	Ti	Al
7.5	0.6	0.25	0.2	0.3	0.07	0.1	0.3	0.04	balance

**Table 2:** The test piece heat treatment plan.

Solution Treatment		Artificial Aging		Combination No
Temperature (*t_s_*), °C	Time (*τ_s_*), h	Temperature (*t_a_*), °C	Time (*τ_a_*), h	
465	0.5	165	2	1
		235	8	2
		325	5	3
	1.5	165	8	4
		235	5	5
		325	2	6
	3	165	5	7
		235	2	8
		325	8	9
520	0.5	165	8	10
		235	5	11
		325	2	12
	1.5	165	5	13
		235	2	14
		325	8	15
	3	165	2	16
		235	8	17
		325	5	18
550	0.5	165	5	19
		235	2	20
		325	8	21
	1.5	165	2	22
		235	8	23
		325	5	24
	3	165	8	25
		235	5	26
		325	2	27

## Data Availability

Data is contained within the article.
